# Evaluation of a New Automated Platform for the Detection and Quantification of HEV RNA

**DOI:** 10.1002/jmv.70923

**Published:** 2026-04-09

**Authors:** Mélanie Lecrac, Sébastien Lhomme, Aurélie Guigon, Benoit Visseaux, Céline Delevallée, Stéphanie Haïm‐Boukobza, Xavier Huc, Annlyse Esteban, Soheil Elhayani, Jacques Izopet, Florence Abravanel

**Affiliations:** ^1^ Hôpital Purpan, Laboratoire de Virologie, Centre National de Référence pour la VHE Toulouse France; ^2^ Inserm UMR 1291 – CNRS UMR5051 – Université de Toulouse, CHU Purpan Toulouse France; ^3^ CHU de Lille, Laboratoire de Virologie Lille Cedex France; ^4^ Département d'Infectiologie Laboratoire Cerba, Cerba Healthcare Frépillon France; ^5^ Laboratoire INOVIE Gen‐Bio Gravanches, 8, rue Jacqueline Auriol Clermont‐Ferrand France; ^6^ Laboratoires Cerballiance, Cerba Healthcare Issy‐Les‐Moulineaux France

**Keywords:** Altostar, Elite Ingenius, Hepatitis E virus, plasma, stool

## Abstract

Hepatitis E virus (HEV) is a major cause of acute hepatitis worldwide. We evaluated the performance of the fully automated HEV RNA assay (HEV ELITe MGB® Kit) using the ELITe InGenius instrument. The limit of detection was established in line with the WHO standard (genotype 3a) and genotype 3f and 3c clinical samples. Finally, the analytical performance of the HEV ELITe MGB® Kit was compared with the Altostar® HEV RNA assay by testing 292 plasma samples and 10 stool samples. The ELITe tool utilized 200 µL of plasma or stool suspension eluted in 100 µL. Linearity was verified from 2 × 10^6^ IU/mL to 2 × 10^3^ IU/mL. The limit of detection for HEV‐3a, HEV‐3c, and HEV‐3f samples ranged from 485 to 523 IU/mL. The qualitative results obtained with both assays were concordant for 276/292 samples (94.5%) and good correlation was observed between both results for positive samples (*ρ* = 0.98, *p* < 0.001). Sixteen samples tested negative with the ELITe assay but were positive using the Altona test, with viral loads ranging from 0.3 to 2.99 log_10_ IU/mL. All HEV RNA were detected in stool samples with both methods. These results indicated a lack of sensitivity for the ELITe assay. A second protocol using 600 µL of sample eluted in 50 µL of solution was evaluated and presented a limit of detection of 69 IU/mL using the WHO standard. While the ELITe assay demonstrated good linearity and correlation with the AltoStar® assay, it struggled to detect low levels of HEV RNA. However, a new extraction protocol may help to overcome this drawback but needs further validation and changes in the package insert of the manufacturer.

## 1 Introduction

Infection with hepatitis E virus (HEV) is a significant cause of morbidity and mortality, representing a considerable global health problem [1]. HEV is a positive‐sense, single‐stranded RNA virus belonging to the *Hepeviridae* family. Of the HEV strains, 4 genotypes mainly affect humans: genotypes 1 and 2 are mainly found in low‐income countries where the virus is usually transmitted by fecal‐oral route through contaminated water and represent a major cause of epidemics. HEV infection is also endemic in developed countries. In such geographical settings, hepatitis E is caused by HEV genotypes 3 and 4, and associated with zoonotic and foodborne transmission. HEV can also be passed on through blood transfusion and organ donation [2].

Clinically, most infections remain asymptomatic or present as generally mild and anicteric disease. HEV usually produces a self‐limiting hepatitis that is clinically indistinguishable from other causes of acute viral hepatitis [2]. HEV infection can induce liver failure and high mortality in patients with underlying chronic liver disease. The mortality rate in pregnant women with genotypes 1 and 2 is approximately 25% [3]. Chronic infection with HEV genotypes 1 and 2 has not been reported so far. However, HEV genotypes 3 and 4 can establish a chronic infection in immunocompromised patients that can ultimately lead to serious complications, including rapid progression to cirrhosis [4, 5].

Virological biomarkers play an important role in the diagnosis, monitoring, and evaluation of antiviral treatment efficacy. The European Association of the Study of the Liver (EASL) guidelines recommend that all patients with symptoms consistent with acute hepatitis should be tested for hepatitis E [6]. They specify that HEV RNA, as well as anti‐HEV IgM are positive markers for acute HEV infection. It is also mandatory to monitor HEV RNA in immunocompromised patients to assess the potential persistence of the virus after 3 months. Detection of HEV RNA in blood and stools is also essential for evaluating the optimal duration of ribavirin therapy for immunocompromised patients [7, 8]. Moreover, as immunocompromised patients are at risk of severe infection, HEV nucleic acid testing (HEV‐NAT) of blood, hematopoietic stem cells, or organ donors has been introduced in several countries. Several automated or semi‐automated HEV RNA instruments are now available for the HEV RNA testing. The Cobas® HEV assay implemented on the Roche instruments (Roche Diagnostics) and Grifols the Procleix HEV RNA assay implemented on the Panther automated platform (Grifols Diagnostic Solutions) provide qualitative assays dedicated to the screening of blood donors [9, 10, 11]. Altostar® HEV RT‐PCR Kit is a quantitative HEV RNA assay provided by Altona Diagnostics [12]. All these assays have a low limit of detection (< 20 IU/mL) assessed with by using the WHO international standard.

We have thus assessed the performance of the fully automated HEV RNA assay (HEV ELITe MGB® Kit) using the ELITe InGenius instrument by following the manufacturer's instructions.

## 2 Material and Methods

Firstly, we have followed the manufacturer's instruction of the HEV ELITe MGB® Kit on the ELITe InGenius system (ELITech group) to assess the analytical performance. Then clinical performance was appraised by testing prospective clinical samples also trialed out using the Altostar® HEV RNA assay on the Altostar AM16 instrument (Altona Diagnostics).

Second, as the sensitivity appears lower than the Altostar® assay, we have evaluated the analytical performance of another extraction protocol based on a higher plasma volume input.

### 2.1 HEV ELITe MGB® Kit

The ELITe InGenius system allows for a single‐step quantitative reverse transcription and a real‐time nucleic acid amplification test to detect and quantify the HEV RNA extracted from clinical samples. The kits also contain an internal control and quantification standards. According to the package insert, this is a specific amplification of the ORF2 region of HEV; it can detect HEV belonging to genotypes 1, 2, 3, 3ra, and 4. The test is validated with plasma collected in EDTA and stool suspensions. The extraction protocol used 200 µL of plasma or stool suspension and RNA was eluted in 100 µL of solution with the The ELITe InGenius SP 200 kit, which is the reagent set for automated DNA/RNA extraction and purification from liquid biological samples. The samples were lysed with a lysis solution in the presence of proteinase K and a nucleic acid carrier. Carrier RNA and an internal control template were added to the samples during the lysis step. RT‐PCR test used 10 µL of extracted RNA. The thermal conditions are retro‐transcription at 50°C for 20 min, then 45 cycles at 95°C for 10 s and 50°C for 35 s.

According to the package insert, the lower limit of quantification (LLOQ) is 288 UI/mL and it is identical to the limit of detection (LOD).

Results were thus interpreted as follows:

‐ “negative” = not detected

‐ “detected not quantifiable” = positive but not quantifiable, below the threshold of 288 IU/mL

‐ “quantifiable” = quantifiable above the threshold of 288 IU/mL.

The assay is linear from 2.45 to 7.27 log_10_ IU/mL.

We also evaluated another extraction protocol on the ELITe InGenius instrument, using the ELITe InGenius SP 1000 kit, which utilized 600 µL of sample eluted in 50 µL. The thermal conditions of the amplification on the ELITe InGenius instrument were similar. This new protocol was tested to assess if it improves the LOD of the HEV ELITe MGB® assay.

### 2.2 AltoStar® HEV RNA Assay

The clinical samples were quantified by using the reference assay AltoStar® HEV RNA Kit in line with the manufacturer's instructions. This semi‐automated test uses 500 µL of sample (plasma or stool suspension) eluted in 80 µL after extraction. RT‐PCR test uses 45 µL of extracted RNA. The thermal conditions are retro‐transcription at 55°C for 20 min, then 45 cycles at 95°C for 15 s and 55°C for 45 s. The LOD is 7.6 IU/mL. The linear range of the AltoStar® HEV RT‐PCR the quantification of HEV in plasma is 5 × 101– 1 × 106 IU/mL [12].

### 2.3 Reproducibility, Limit of Detection, Range of Linearity

The intrarun reproducibility was assessed by testing five replicates of the same positive sample during a single run. The interrun variability was assessed by testing five replicates of the positive sample in five different runs. The variability of the HEV ELITe MGB® Kit was estimated with the standard deviation of the log_10_ HEV RNA values obtained.

In order to determine the limit of detection and the linearity of the HEV ELITe MGB® Kit, we used the 1st WHO reference standard (PEI code 6329/10, genotype 3a) and 2 clinical samples containing HEV genotypes 3c and 3f, which are the two major genotypes found in Europe [13]. The clinical samples were previously quantified through the AltoStar® assay, and this quantification was verified with our home‐brew assay [14].

To calculate the LOD using the Probit method, we tested samples diluted in negative plasma at 5 HEV RNA concentrations (1000, 500, 250, 125, and 62.5 IU/mL), and 10 replicates were analyzed for each concentration.

The linearity domain of the HEV ELITe MGB® Kit was carried out by testing dilutions of a clinical strain of genotype 3c previously quantified with the Altostar® system. Those samples and dilutions were tested in triplicates in the same run and the results were compared to the expected values.

### 2.4 Method Comparison

We conducted a prospective comparative study on fresh stool and plasma samples received in our laboratory, which acts as the French National Reference Centre for HEV. 292 plasma samples were taken with EDTA and 10 stool samples with the Altostar® assay, enabling a total of 302 tests to be carried out over 3 months via the ELITe system and HEV ELITe MGB® Kit. The samples were sent to our laboratory for HEV diagnosis, HEV monitoring, or HEV RNA confirmation and genotyping. One replicate of each sample with both assays was performed for the evaluation.

### 2.5 Statistical Analyses

HEV RNA values were log‐transformed and then analyzed with XLStat software. The Spearman test was applied to check for any correlation between the two assays. *P*‐values lower than 0.05 were considered statistically significant. A Bland–Altman analysis (a scatter plot of the differences between the paired measurements plotted against their means) was used on samples that tested positive with both methods to assess the magnitude of disagreement between them and to estimate the overall bias.

## 3 Results

### 3.1 Performance of the HEV ELITe MGB® Kit Following the Manufacturer's Instructions

#### 3.1.1 Analytical Performance

The intrarun reproducibility of the HEV ELITe MGB® Kit, estimated with the standard deviation of the log HEV RNA values obtained for a positive sample was 0.05 IU/mL (Table 1). The interrun reproducibility of the HEV ELITe MGB® Kit, estimated with the standard deviation of the log HEV RNA values obtained for the positive control (HEV RNA concentration 5.08 log IU/mL), was 0.08 IU/mL for the positive control tested on five replicates in different runs (Table 2).

**Table 1:** Intrarun varibility of a positive sample.

	HEV RNA concentration (log_10_ IU/mL)
Replicate #1	3.99
Replicate #2	3.96
Replicate #3	4.05
Replicate #4	3.97
Replicate #5	3.92
Mean	3.98
Standard deviation	0.05

**Table 2:** Interrun varibility of the positive control.

	HEV RNA concentration (log_10_ IU/mL)
Replicate #1	5.07
Replicate #2	4.96
Replicate #3	5.12
Replicate #4	5.15
Replicate #5	5.12
Mean	5.08
Standard Deviation	0.08

We used the international standard provided by the Paul Erhlich Institute (code 6329/10, genotype 3a) and two clinical samples to assess the limit of detection by Probit regression analysis (Table 3). The LOD determined for the HEV ELITe MGB® Kit was 485 IU/mL (95% confidence interval [CI]: 338–1257 IU/mL) for the WHO standard, 523 IU/mL (95% CI: 355–1637 IU/mL) for the HEV‐3c sample and 500 IU/mL (95% CI: 330–2180 IU/mL) for the HEV‐3f sample.

**Table 3:** Limit of detection of the HEV ELITe MGB® Kit assay.

Genotypes	HEV concentration (IU/mL)	% positive (*N* positives/*N* valid replicates)
Genotype 3a (WHO standard)	1000	100 (10/10)
	500	100 (10/10)
	250	50 (5/10)
	125	70 (7/10)
	62.5	30 (3/10)
Genotype 3c	1000	100 (10/10)
	500	90 (9/10)
	250	80 (8/10)
	125	60 (6/10)
	62.5	30 (3/10)
Genotype 3f	1000	100 (10/10)
	500	90 (9/10)
	250	100 (10/10)
	125	100 (10/10)
	62.5	40 (4/10)

**Figure 1:**
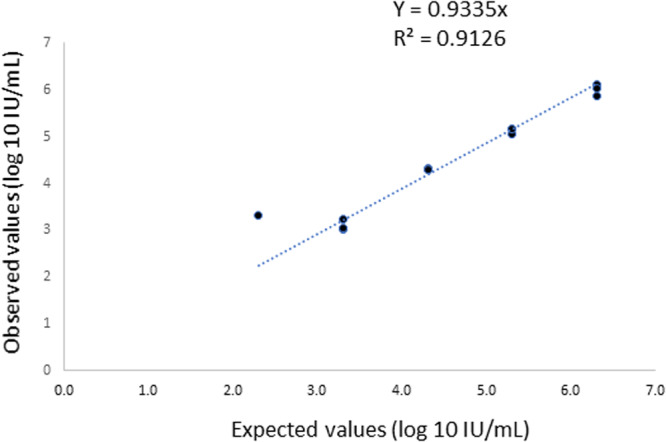
Linearity of ELITech instrument of a positive plasma quantified at 2 × 10^6^ log_10_ IU/mL.

The linearity of the ELITe assay was rated by testing 10‐fold serial dilutions of positive plasma quantified at 2 × 10^6^ log_10_ IU/mL in triplicate (Figure 1). The measurements of two out of the three samples at 2 × 10^2^ IU/mL were found to be “detected not quantified” (result < 288 UI/mL). Thus, linearity was verified from 2 × 10^6^ to 2 × 10^3^ IU/mL.

#### 3.1.2 Clinical Performance of the HEV ELITe MGB® Kit by Comparison With the Altostar® Assay

We compared the two automated assays, the HEV ELITe MGB® Kit and the Altostar® HEV RNA through a prospective study, using fresh plasma to investigate the concordance and correlation between them. We also tested 10 stool samples.

The qualitative results obtained with the two assays were concordant for 276/292 samples (94.5%) (Table 4). One hundred and seventy samples were negative, and 106 were positive with both assays. Among the concordant results, 25 positive samples from the Altona test, with a median viral load of 2.63 log_10_ IU/mL (range: 1.76−3.16 log_10_ IU/mL), were tested and turned out to be “detected not quantified” with the ELITe test. The Ct values obtained with ELITe assay ranged from 38.16 to 41.9 for these 25 samples HEV RNA positive plasma. One sample with a HEV RNA concentration of 8 log_10_ IU/mL with Altonastar®, had for result > 7.27 log_10_ IU/mL with the ELITe assay.

**Table 4:** Agreement between Altona and Elitech results.

		Altona		
Elitech		Positive	Negative	TOTAL
	Positive	81	0	81
	Detected not quantifiable	25	0	25
	Negative	16	170	186
	Total	122	170	292

Sixteen samples out of 292 were clearly discrepant, they tested negative with the ELITe assay but positive using Altona test; viral loads ranged from 0.3 to 2.99 log_10_ IU/mL when employing the Altona test. Lastly, no samples were negative in Altona and positive in ELITe

**Figure 2:**
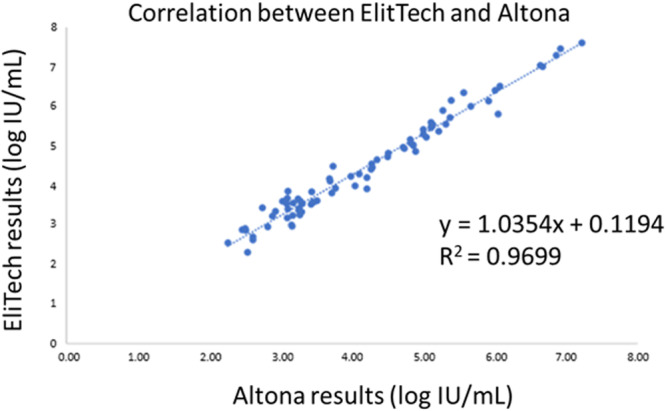
Correlation between ELITech and Altona HEV RNA measurement in plasma illustrated by Deming regression.

**Figure 3:**
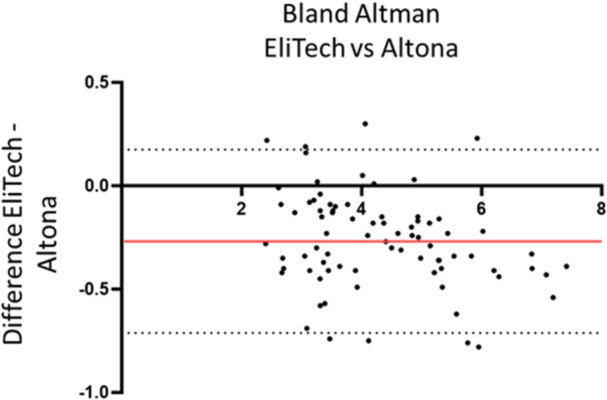
Assessment of the bias between ELITech and Altona through Bland Altman analysis. The red line represents the mean difference, and dashed lines represent the 95% confidence intervals.

The correlation was estimated for the positive samples quantified in log_10_ IU/mL with both assays. The 25 samples “detected not quantified” with the ELITe test, and the samples with a result > 7.27 log_10_ IU/mL with the ELITe assay couldn't be included in the analysis. Thus, 80/106 quantified results with both assays were analyzed. The assays were strongly correlated (Spearman□ correlation coefficient: *ρ *= 0.98, *p* < 0.001). Figure 2 illustrates the correlation between the Altostar® values and the ELITe values. The Bland‐Altman analysis in Figure 3 illustrates the mean difference between the two assays. Bland Altman analysis revealed a slight overestimation of 0.26 log_10_ IU/mL for the Altostar® assay (Figure 3).

The 10 stool samples tested positive with the Altostar® and ELITe methods.

### 3.2 Evaluation of a Different Extraction Protocol

The analytical performance of the ELITe assay was studied with a different extraction protocol on the ELITe InGenius system: 600 µL of samples eluted in 50 µL, instead of 200 µL eluted in 100 µL.

The intrarun reproducibility of the HEV ELITe MGB® Kit, estimated with the standard deviation of the log HEV RNA values obtained for a positive sample was 0.05 IU/mL. The interrun reproducibility of the HEV ELITe MGB® Kit, was 0.06 IU/mL (Table 5).

**Table 5:** intrarun and interrun variability with the new extraction protocol.

	Intrarun variability	Interrun variability
	HEV RNA concentration (log_10_ IU/mL)	HEV RNA concentration (log_10_ IU/mL)
Replicate #1	3.85	3.29
Replicate #2	3.87	3.29
Replicate #3	3.96	3.16
Replicate #4	3.93	3.26
Replicate #5	3.97	3.29
Mean	3.92	3.26
Standard deviation	0.05	0.06

The limit of detection of the ELITe assay with this different extraction protocol was studied by testing serial dilutions of the WHO international standard. Ten replicates per concentrations of 1000, 500, 250, 125, 62.5, and 31.25 IU/mL were prepared and tested (Table 6). Probit analysis found a LOD of 69 IU/mL (95% CI: 54–170 IU/mL) with this new protocol.

**Table 6:** Limit of detection of the HEV ELITe MGB® Kit assay with a new extraction protocol.

Genotypes	WHO standard HEV concentration (IU/mL)	% positive (*N* positives/*N* valid replicates)
Genotype 3a	1000	100 (10/10)
	500	100 (10/10)
	250	100 10/10)
	125	100 (10/10)
	62.5	90 (9/10)
	31.25	30 (3/10)

**Figure 4:**
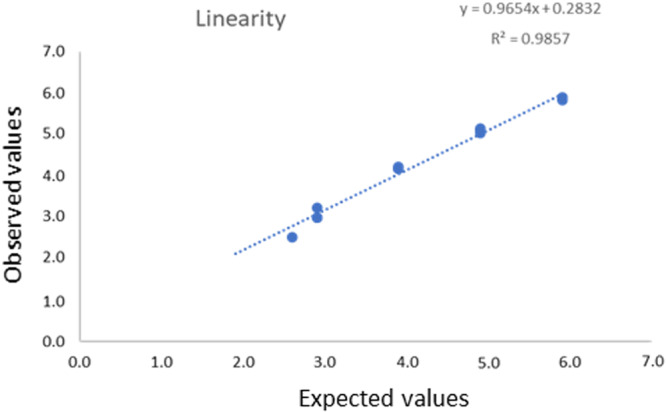
Linearity of the ELITech Instrument with a positive plasma using the new protocol.

Linearity was assessed by testing 10‐fold serial dilutions of a genotype 3c sample from 5.9 to 1.9 log_10_ IU/mL and verified from 5.9 to 2.9 log_10_ IU/mL (Figure 4).

## 4 Discussion

We assessed the analytical and clinical performance of the HEV ELITe MGB® Kit according to the manufacturer's instructions. This evaluation indicated a lack of sensitivity of the assay. However, our preliminary analytical evaluation of a new extraction protocol could overcome this drawback.

The ELITe assay presented good linearity from 2.6 to 5.9 log_10_ IU/mL. This aligns with the linearity of the automated Altona platform used as a comparator. In our previous evaluation, the Altostar assay was linear from 2 to 6 log_10_ IU/mL [12]. The linearity of the ELIite assay was also verified with another improved extraction protocol. The limit of detection, assessed with the WHO international standard or clinical samples and the classic extraction protocol, indicated a LOD of HEV RNA in plasma at around 500 IU/mL. The difference in LOD claimed in the package insert (288 UI/mL) may be explained by separate experiments conducted with a different range of serial dilutions.

This LOD is 10‐fold higher than the LOD observed for other automated platforms that found LOD < 50 IU/m [9, 11, 12, 15]. A low LOD may help to detect HEV RNA for anti‐HEV IgM‐positive patients with low virus loads [9]. This lower sensitivity of the ELITe assay was also observed on plasma clinical samples. Sixteen samples were negative with the ELITe assay but positive using the Altostar® test, with viral loads ranging from 0.3 to 2.99 log_10_ IU/mL for the Altona test. These 16 samples were collected in patients under ribavirin therapy with declining HEV RNA concentration (*n* = 4) or in blood donors (*n* = 12) that tested HEV RNA positive with the Cobas HEV kit, whose LOD is 11.76 (95% CI, 8.58–18.97) IU/mL [15]. Additionally, 25 positive samples tested positive but were not quantified with the ELITe test (< 288 IU/mL). Similarly, these 25 samples were collected in patients under ribavirin therapy or in blood donors (*n* = 12). The quantitative results expressed in IU/mL for both assays were also consistent with a limited bias between the two assays. Thus, discrepant results between the two assays observed in clinical samples are due to the lower sensitivity of the ELITe assay.

HEV RNA monitoring is crucial in immunocompromised patients for assessing chronic HEV infections or initiating anti‐viral therapy [6]. The ELITe assay may have a lower sensitivity for detecting low HEV RNA concentrations. However, another protocol using 600 µL of plasma was evaluated and improved the limit of detection. This different extraction protocol is validated for the detection of HIV‐1 RNA on the ELITe InGenius system with a LOD of 26 copies/mL [16].

Interestingly, the ELITe assay is also suitable for detecting HEV RNA in stool samples. This matrix may be more challenging for molecular assays since they contain PCR inhibitors [17, 18]. However, it is essential to monitor the HEV RNA in stools to assess the risk of relapse in HEV‐infected patients under ribavirin therapy and optimize treatment duration [7, 8].

In terms of instrumentation, the ELITe reagents are not fully ready to use unlike in the Altostar® assay. The ELITe Ingenius system has an analysis time of 2.5 h for 12 samples, while the Altostar instrument undertakes the analysis of 96 samples in 6 h. The ELITe assay requires a calibration every 2 months compared to each run for the Altostar® assay. These differences in throughput offer medical biology laboratories new options adapted to their specific needs in terms of sample volumes to be processed. Additionally, the ELITech group now provides several molecular assays incorporating the ELITe InGenius for microbiology diagnosis, and other commercial or laboratory‐developed assays can be implemented on this tool [19, 20].

One limitation of this study is that we did not evaluate the new extraction protocol on clinical samples since this would have required a bigger volume of samples that we could not obtain for the current prospective study. Thus, the further evaluations of the clinical performance, especially on samples containing a low viral load are required to validate this modification of the manufacturer's instructions. The genotype of the samples was not determined as some had very low viral load although the vast majority (> 99%) of the HEV strains characterized in our laboratory belonged to genotype 3. The LOD for genotype 3f and 3c and the linearity of the Elite assay was assessed with samples previously quantified with the Altostar® assay and not with samples from international panel. However, the Altostar® assay uses four standards of quantification calibrated against the 1st WHO International Standard for HEV RNA for nucleic acid amplification techniques (PEI code: 6329/10). Moreover, the results of LOD obtained for the genotype 3c and 3f samples agrees with the LOD obtained for the WHO standard (genotype 3a, PEI code: 6329/10).

In conclusion, the HEV RNA ELITe InGenius assay produces precise quantification of the viral load in plasma and performs accurate detection in stools. The low limit of detection in plasma illustrated in the current protocol may be overcome by a new extraction protocol that uses a larger volume of plasma. But, the clinical validation of this different extraction protocol for HEV RNA warrants further evaluation and changes in the package insert of the manufacturer.

## Author Contributions

**Mélanie Lecrac:** writing – original draft, data curation. **Florence Abravanel:** writing – original draft, validation, supervision. **Aurélie Guigon:** data curation. **Sebastien Lhomme:** writing – review and editing, data curation. **El Hayani Soheil:** formal analysis. **Annlyse Esteban:** formal analysis. **Benoit Visseaux:** writing – review and editing, conceptualization. **Céline Delevallée:** writing – review and editing, conceptualization. **Stéphanie Haïm‐Boukobza:** qriting – review and editing, conceptualization. **Xavier Huc** and **Jacques Izopet:** writing – review and editing, conceptualization.

## Conflicts of Interest

The authors declare no conflicts of interest.

## Data Availability

The data that support the findings of this study are available from the corresponding author upon reasonable request.
